# Narcissistic Traits and Executive Functions

**DOI:** 10.3389/fpsyg.2021.707887

**Published:** 2021-11-01

**Authors:** Igor Nenadić

**Affiliations:** ^1^Department of Psychiatry and Psychotherapy, Philipps Universität Marburg, Marburg, Germany; ^2^Department of Psychology, Goethe-Universität Frankfurt, Frankfurt, Germany; ^3^Department of Psychiatry and Psychotherapy, Jena University Hospital, Jena, Germany

**Keywords:** narcissistic traits, narcissism, working memory, executive functions, subclinical, schema therapy

## Abstract

Several personality disorders have been associated with cognitive impairment, including executive functions like working memory. Yet, it is unclear whether subclinical expression in non-clinical persons is associated with cognitive functioning. Recent studies indicate that non-clinical subjects might, in fact, perform better with increasing moderate to mild expressions of narcissistic features. We tested working memory performance in a cohort of *n*=70 psychiatrically and neurologically healthy subjects using Wechsler Adult Intelligence Scale (WAIS/WIE) subtests Arithmetic, Digit Span and Letter-Number Sequencing, and assessed narcissistic features using three different inventories: the widely used Narcissistic Personality Inventory (NPI), as well as two clinically used measures of narcissistic traits and states, respectively, derived from schema-focused therapy, i.e., the Young Schema Questionnaire (YSQ) entitlement/grandiosity subscale and the Schema Mode Inventory (SMI) self-aggrandizer subscale. In accordance with our hypothesis, we found nominally significant positive correlations of WIE Arithmetic performance with NPI total score (Spearman’s rho=0.208; *p*=0.043) and SMI self-aggrandizer scale (Spearman’s rho=0.231; *p*=0.027), but findings did not survive false discovery rate (FDR) adjustment for multiple comparisons (p_FDR_=0.189 and p_FDR_=0.243, respectively). While our findings add to recent studies on cognitive performance in subclinical narcissism, they fail to demonstrate an association of cognitive performance with narcissistic traits across multiple working memory tests, indicating the need for additional study, including complementary executive functions in larger cohorts and ranges of phenotype expression.

## Introduction

Personality disorders have increasingly been associated with impairments in cognitive performance, including executive functions like working memory, inhibition, flexibility or decision-making (Garcia-Villamisar et al., 2017). This is not only the case for cluster A personality disorders like schizotypal personality disorder (Siddi et al., 2017), which have typically been studied as minor expressions of psychosis-like disorders, or related personality traits like schizotypy, often used as a marker of psychosis proneness (Ettinger et al., 2015).

While initial studies have suggested cognitive impairment in cluster B personality disorders/traits (‘dramatic personalities’; Burgess, 1992), an increasing number of more recent studies has substantiated and expanded on these findings, particularly on borderline personality disorder (e.g., Nemeth et al., 2020; for review, see McClure et al., 2016), but also antisocial/psychopathy and narcissistic personality features (see overview in Garcia-Villamisar et al., 2017) and even (cluster C) obsessive–compulsive personality traits (Garcia-Villamisar and Dattilo, 2015).

Among the studied executive functions is working memory, which refers to an individual’s ability to maintain a limited amount of information over a short span, usually a few seconds and has been linked to prefrontal-subcortical brain circuitry functioning (Baddeley, 2012; D’Esposito and Postle, 2015; Chai et al., 2018; Miller et al., 2018b).

Narcissistic personality disorders (NaPD) or personality features have received less attention in this field. While studies in patients with NaPD are still scarce, there are some recent findings on narcissistic personality traits, which can be used to conceptualise a spectrum or continuum ranging from subclinical expressions of the narcissism phenotype to manifest NaPD (Blais and Little, 2010; Miller and Campbell, 2010; Wright et al., 2013).

Two lines of evidence suggest that narcissistic traits affect cognitive performance and functioning. First, studies in patients with (other) neuropsychiatric disorders have suggested that the extent to which these patients display narcissistic features might affect cognitive impairment. For example, in a cohort of patients with juvenile myoclonus epilepsy, narcissistic traits [assessed using the Personality Belief Questionnaire (PBQ), which is a self-assessment inventory oriented towards DSM-IV (American Psychiatric Association, 2000) personality disorder categories, and thus more grandiose facets] were negatively correlated with TMT-A measures of processing speed and Stroop measures of executive function (Taura et al., 2020), and similarly in schizophrenia spectrum, disorders rates of narcissistic traits [assessed using the Millon Clinical Multiaxial Inventory (MCMI-III), again based on DSM-IV clinical narcissism facets] correlated positively with processing speed and executive function in the Bell-Lysaker Emotional Recognition Task, Wisconsin Card Sorting Text, WCST, and Continuous Performance Test, CPT (Lysaker et al., 2004). However, in a patient group with closed head injury, narcissistic features from the MCMI-III were positively correlated with processing speed (TMT-A and Stroop Words and Colours) and language function (Boston Naming Test, WAIS-III Vocabulary, Comprehension and Similarity subtests; Ruocco and Swirsky-Sacchetti, 2007), raising the issue of directionality of effects, which might vary across tasks or underlying brain disorders.

In a second group of studies, neurologically and psychiatrically healthy subjects with varying levels of narcissistic traits (mostly in modest to moderate ranges) were assessed using neuropsychological tasks. Studies on complex decision-making tasks have failed to provide consistent support for an association with narcissistic traits (NPI) or facets of pathological narcissism (NARQ scores; Brunell and Buelow, 2015; Buelow and Brunell, 2020).

In addition, more general facets of cognitive functioning, such as intelligence, have been studied in an adolescent cohort, identifying a significant positive correlation of grandiose narcissism (assessed by the NPI) with performance on a modified Raven’s test as a measure of objective intelligence (Zajenkowski, 2021), as well as in student samples, identifying a positive correlation of grandiose narcissism with subjective intelligence [but not objectively assessed intelligence using Cattell’s Culture Fair Intelligence Test (CFIT; Zajenkowski et al., 2020)].

While the studies in clinical cohorts (see above) have tended to use clinical indicators of narcissism (e.g., MCMI), often related to DSM-IV criteria of NaPD (American Psychiatric Association, 2000), studies in non-clinical cohorts have often used NPI, which is generally considered to reflect the grandiose and entitlement facets of narcissism, rather than vulnerable narcissism or covert narcissism (for review of facets, see Pincus and Lukowitsky, 2010; Pincus et al., 2014; Ackerman et al., 2019).

In a recent study, however, Zhang and colleagues demonstrated in a non-clinical cohort that narcissistic traits are indeed associated with better performance on a cognitive task (Zhang et al., 2020), at least when adaptive and maladaptive narcissisms interact; more interestingly, in the subclinical range of minor or modest expression of a narcissistic phenotype, there seems to be a positive (rather than negative) correlation, which might contradict the notion of a linear continuum. In the case of processing particular emotional stimuli salient to narcissists, higher narcissistic features are even associated with faster performance at accurately identifying certain emotional stimuli (De Panfilis et al., 2019), whereas emotion recognition in clinical NaPD appears to be impaired (Marissen et al., 2012).

The current literature on cognitive performance and narcissistic traits therefore has some shortcomings, including lack of studies specifically addressing working memory functions, which could be related of prefrontal neural circuitry (D’Esposito and Postle, 2015; Lara and Wallis, 2015), and also the use of different narcissism inventories, derived mostly from social psychological research rather than clinical ones. Given recent neuroimaging studies linking trait narcissistic features to prefrontal brain structure (Mao et al., 2016), as well as prefronto-parietal brain activity (Yang et al., 2015), which are relevant to executive functions like working memory, cognitive studies might help to elucidate the functional impact of these traits in the non-clinical (or subclinical) range of a putative narcissism spectrum (Miller and Campbell, 2010).

In the present study, we aimed to specifically test the hypothesis that narcissistic traits in a non-clinical population are associated with better working memory performance. Aimed at the non-clinical part of the putative narcissism spectrum, our study aimed to use both the narcissistic personality inventory (NPI) as a well-established indicator of narcissistic traits, as well as additional measures derived from clinical practice. For the latter, we chose trait and state markers commonly used in diagnostic assessment of personality disorder patients based on the schema focused therapy (or schema therapy) framework introduced by Young et al. (2003), which are aimed at capturing a narcissistic-like phenotype commonly observed in personality disorders and subclinical expressions. This approach to narcissistic features is based on biographical development of maladaptive cognitions, emotional styles and behaviours in the context of ‘impaired limits’ in interaction and is thought to mostly reflect grandiose self-image and entitlement (based on parental over-gratification), for details see also Young et al. (2003), Lobbestael et al. (2010), Bach et al. (2018).

## Materials and Methods

### Study Cohort

We included *n*=70 clinically healthy subjects (34 female, 36 male; mean age 31years, SD 10.9) for this study. Subjects were drawn from healthy control samples of ongoing projects at the Department of Psychiatry and Psychotherapy of Jena University Hospital, overlapping with a cohort used for a patient case–control study of cognition in psychiatric disorders (Nenadic et al., 2015b). Exclusion criteria for this cohort included psychiatric or neurological disorders (including traumatic brain injury with loss of consciousness), or previous or concurrent psychotherapy or medication treatment with psychotropic drugs, as well as lack of alcohol or illicit drug dependence, all established through semi-structure screening protocols with self-report prior to inclusion into the study. In addition, none of the subjects had a first-degree relative with a psychotic disorder.

All participants gave written informed consent to study protocol approved by the Ethics Committee of the Medical School of Friedrich-Schiller-University of Jena (protocol approval numbers 2077-05/07, 2641-08/09 and 3144-05/11).

In order to assess general IQ, the German MWT-B (Mehrfachwahl-Wortschatz-Test B) was used (Lehrl et al., 1995; Lehrl, 2005), a IQ proxy similar to the National Adult Reading Test, NART. Handedness was assessed by self-report using Oldfield’s Edinburgh Handedness Scale, EHI (Oldfield, 1971).

### Psychometric Assessment

We used three different self-report questionnaires/scales to assess facets of narcissism in this sample: the NPI and narcissism-related subscales from two inventories commonly used in schema therapy diagnostics: the Young Schema Questionnaire (YSQ) and the Schema Mode Inventory (SMI).

First, we administered the NPI (Raskin and Hall, 1979), one of the most widely used narcissism inventories, especially in social psychology, but also in clinical studies (for review/meta-analyses, see Grijalva et al., 2015; Grijalva and Zhang, 2016). While initial studies suggested seven first-order components designated authority, exhibitionism, superiority, vanity, exploitativeness, entitlement and self-sufficiency (Raskin and Terry, 1988), there has been a plethora of subsequent studies discussing the factor structure of the NPI across different cohorts and translations (Emmons, 1984; Kansi, 2003; Barelds and Dijkstra, 2010; Ackerman et al., 2011; Braun et al., 2016; Dinic and Vujic, 2019), also with regards to binary vs. Likert scale response variants (Boldero et al., 2015; Miller et al., 2018a,c), cross-cultural aspects (Zemojtel-Piotrowska et al., 2019), centrality/network structure (Briganti and Linkowski, 2020) and the phenotype it characterises. We administered the NPI, using the German long version (40 items, forced-choice response format), which had been validated and studied in four large German samples, and from which the above subscales can be extracted (Schütz et al., 2004). Sample items (forced-choice decision of pairs) include ‘I have a natural talent for influencing people’ vs. ‘I am not good at influencing people’ or ‘I am an extraordinary person’ vs. ‘I am much like everybody else’. For NPI, Cronbach’s alpha in German reference samples was 0.82, with retest reliability of 0.89 (Schütz et al., 2004).

Second, we used the entitlement/grandiosity subscale from the YSQ, applying the validated German version of the YSQ-S2, short version 2 (Grutschpalk, 2008; Roediger, 2011; Wichmann, 2012) as used in a previous study of ours (Nenadic et al., 2020a,b). The YSQ was developed to assess early maladaptive schemas (EMS), a construct central to schema therapy (Young et al., 2003), a psychotherapy approach building on cognitive-behavioural therapy for the treatment of personality disorders. Within the schema therapy model, EMS denote trait-like markers of personality and therefore provide a clinically useful validated measure of interpersonally challenging behaviours (for meta-analyses, see Bach et al., 2018; Janovsky et al., 2020). Sample items include: ‘I feel that I shouldn’t have to follow the normal rules or conventions that other people do’ and ‘I have difficulties accepting a “no” when demanding something from others’. For YSQ-S2 entitlement/grandiosity subscale, Cronbach’s alpha in German reference samples was 0.79 (Siegmund et al., 2011) and 0.70 (Wichmann, 2012), respectively.

Third, we also used the ‘self-aggrandizer’ subscale from the SMI 1.1 in its German version (Roediger, 2011; see also Dominiak, 2014) and for psychometric evaluation (Lobbestael et al., 2010). In the theory and practice of schema therapy (Young et al., 2003), schema modes (or modes) are conceptualised as a current cognitive-emotional state of a subject and as the current realisation of an EMS-related reaction in a given situation. As such, SMI scales rather reflect state-like components. However, the repeated occurrence of these modes in the sense of dysfunctional coping with internal emotional states also reflects a frequency of occurrence in the individuals’ behaviour. The coping modes within the SMI can be organised into coping styles related submission, avoidance or overcompensation (analogous to the freeze, flight and fight reactions), and we chose the ‘self-aggrandizer’ as it reflects a state commonly encountered in narcissistic patients showing grandiose, entitled overcompensation in difficult emotional states (Young et al., 2003). Hence, while this variable is not specific to narcissism, it is a behavioural parameter which can be assumed to be linked to narcissistic behaviour, which is also substantiated by its correlation with narcissism measures, such as the entitlement/grandiosity subscale of the YSQ. Sample items include as: ‘I get irritated when people don’t do what I ask them to do’ or ‘I feel special and better than most other people’. For SMI subscale self-aggrandizer, Cronbach’s alpha in initial samples was 0.83 (Lobbestael et al., 2010) and 0.074 in a German reference sample including patients and non-clinical subjects (Reiss et al., 2012).

### Neuropsychological Assessment

Subjects underwent neurocognitive assessment for working memory/executive functions, mostly taken from the Wechsler Intelligenztest für Erwachsene [WIE, the German version of the Wechsler Adult Intelligence Scale (WAIS-IIIR)], as part of a previously published neurocognitive battery initially devised for a twin cognition study (Goldberg et al., 2013; Nenadic et al., 2015b).

As measures of working memory/executive function, we used the Arithmetic, Digit Span and Letter-Number-Sequencing tasks from the WIE (which form the working memory index of the WIE/WAIS-IIIR).

In addition, the present study also provides an exploratory analysis of additional neuropsychological tests, as the cognitive battery assessed in these subjects was designed for use in other studies, and thus included tests of other cognitive domains. In particular, we also administered from the WIE: the digit symbol test (part of the WIE processing speed index group), matrix reasoning (perceptual organisation index group) and information task (verbal comprehension index group). In addition to the WIE tests, subjects also received the Trail Making Tests (TMT-A and TMT-B), in which the TMT-B also includes a significant inhibition and thus executive functioning component. As variables, we used both the duration for test completion (in seconds) for the TMT-A and TMT-B, as well as the difference (subtracting TMT-A from TMT-B) as a means of a more specific executive functioning parameter, which ‘removes’ the processing speed component reflected in TMT-A duration. Finally, the Controlled Oral Word Association (COWA) task was administered, asking subjects to produce as many nouns starting with the letters F, A and S, respectively, each during 1min (i.e., three runs, one for each letter) or naming animals, respectively. For descriptions of neuropsychological tests, see also Lezak et al. (2012).

### Statistical Analysis

We initially used G*Power 3.1 for computation of required sample size based on G*Power’s model for exact tests and correlation analyses. Assuming alpha error probability of 0.05 and power of 80%, as well as correlation of 0.3 and one-tailed testing (based on the study’s directed hypothesis), G*power estimated a necessary sample size of 67 subjects (note that the same calculation with two-tailed testing resulted in a necessary sample size of 84 subjects).

For statistical analysis, we used SPSS (version 25; IBM). Prior to hypothesis testing, we used non-parametric correlations (Spearman’s rho) to test for correlations between the NPI total, YSQ-entitlement/grandiosity and SMI self-aggrandizer scores.

We tested our study’s main hypothesis using non-parametric correlations (Spearman’s rho) between each of these three narcissism-related variables and the three working memory-related WIE tests (Arithmetic, Digit Span and Letter-Number-Sequencing tasks) as well as the TMT-B. The False Discovery Rate (FDR) approach was used to adjust for multiple comparisons in hypothesis-led analyses (Benjamini and Hochberg, 1995).

In addition to this testing of primary hypotheses, we performed exploratory analysis for NPI total score, YSQ-entitlement/grandiosity and SMI self-aggrandizer scores with other neuropsychological tests obtained in this battery not directly related to working memory (i.e., WIE digit symbol, WIE matrix reasoning and WIE information scores, TMT-A and TMT-B, and COWA scores), as a means to explore associations of narcissistic traits with other cognitive domains.

## Results

We found nominally significant positive correlations of the WIE arithmetic task with both NPI total score (Spearman’s rho=0.208; *p*=0.042) and SMI self-aggrandizer scale (Spearman’s rho=0.231; *p*=0.027), but not YSQ entitlement scale (Spearman’s rho=0.089; *p*=0.231); as well as a trend-level correlation of SMI self-aggrandizer scale with WIE Letter-Number Sequencing (Spearman’s rho=0.166; *p*=0.085); for overview, see Table 1. FDR-adjusted values, however, indicated that none of these correlations survived adjustment for multiple comparisons (FDR-adjusted values of *p* for above correlations: 0.189; 0243; 0.3465; and 0.255 for the trend-level finding, respectively).

**Table 1:** Correlation analysis of narcissism measures narcissistic personality inventory (NPI, 40-item version), YSQS2 entitlement scale and SMI self-aggrandizer scale with working memory scores of Wechsler Adult Intelligence Scale (WIE) in *n*=70 psychiatrically healthy subjects.

		NPI total	YSQ2 entitlement	SMI self-aggrandizer
Arithmetic (WIE)	Spearman’s rho (*p*)	0.208^*^ **(0.042)**^†^	0.089 (0.231)	0.231^*^ **(0.027)**^††^
Digit span (WIE)	Spearman’s rho (*p*)	0.139 (0.125)	−0.041 (0.369)	0.083 (0.247)
Letter-Number sequencing (WIE)	Spearman’s rho (*p*)	0.142 (0.120)	−0.046 (0.352)	0.166 (0.085)

* Statistical significance.

† *FDR-adjusted value of p: 0.189*.

†† *FDR-adjusted value of p: 0.243*.

*Bold indicates nominally significant (p<0.05) correlations and underlined values indicate trends; and none of these correlations survived FDR adjustment for multiple comparisons*.

In our exploratory analyses of other cognitive measures (see Table 2), we also observed correlations of NPI total score and SMI self-aggrandized scales with WIE matrix reasoning (Spearman’s rho=0.202; *p*=0.047; and Spearman’s rho=0.260; *p*=0.015, respectively), as well as the SMI self-aggrandized scale with the WIE information test (Spearman’s rho=0.289; *p*=0.008; for full overview, see Table 2), but none of these findings remained significant after FDR adjustment for multiple comparisons.

**Table 2:** Exploratory correlation analysis of narcissism measures NPI (narcissistic personality inventory, 40-item version), YSQS2 entitlement scale and SMI self-aggrandizer scale with other cognitive measures, not directly/specifically tapping working memory.

		NPI total	YSQ2 entitlement	SMI self-aggrandizer
Digit symbol (WIE)	Spearman’s rho (*p*)	0.067 (0.292)	−0.024 (0.421)	0.067 (0.292)
Matrix reasoning (WIE)	Spearman’s rho (*p*)	0.202 (0.047)^*^	0.010 (0.467)	0.260 (0.015)^*^
Information task (WIE)	Spearman’s rho (*p*)	0.164 (0.088)	−0.091 (0.226)	0.289 (0.008)^*^
Trail Making Test A (TMT-A)	Spearman’s rho (*p*)	−0.174 (0.074)	0.146 (0.113)	−0.035 (0.384)
Trail Making Test B (TMT-B)	Spearman’s rho (*p*)	−0.160 (0.093)	0.036 (0.384)	−0.092 (0.226)
TMT-B – TMT-A difference	Spearman’s rho (*p*)	−0.071 (0.279)	−0.058 (0.315)	−0.086 (0.241)
COWA letters	Spearman’s rho (*p*)	0.101 (0.203)	−0.192 (0.055)	−0.052 (0.336)
COWA animals	Spearman’s rho (*p*)	−0.012 (0.460)	−0.142 (0.120)	−0.116 (0.169)

* *(nominally) significant at p<0.05*. Bold script indicates nominal significance.

Across our applied inventories, the NPI total scale was significantly correlated with YSQ-entitlement/grandiosity (Spearman’s rho=0.239; *p*=0.023), but particularly the SMI self-aggrandizer scale (Spearman’s rho=0.5; *p*<0.001); while YSQ-entitlement/grandiosity and SMI self-aggrandizer scales were also highly correlated (Spearman’s rho=0.489; *p*<0.001).

## Discussion

Our study did not find evidence of a statistically significant correlation (following correction for multiple comparisons) between measures of executive function/working memory and either the NPI or the two schema therapy-based indicators of narcissistic traits/states, i.e., YSQ and SMI. When considering uncorrected findings (i.e., ‘nominally significant’ findings, prior to correction for multiple comparison), we identified only very limited evidence for a potential association of narcissistic traits with working memory performance in a cohort of psychiatrically healthy subjects, but these positive correlations between WIE working memory scores and NPI and SMI (a state indicator of narcissistic-like derived from clinical practice) did not survive FDR adjustment for multiple comparisons and thus cannot be considered a reliable indicator of a correlation of working memory with narcissistic traits.

While the nominally significant results are partially in line with our hypotheses, the overall negative findings of our study raise some potentially relevant issues in understanding cognitive function in the narcissism spectrum and approaching the problem in future studies.

In interpreting our findings against the literature on narcissism, three aspects seem to merit particular attention for explaining the overall negative outcome of our study: first, the lack of (refined) neuroanatomical models of narcissism (either clinical or non-clinical) that would allow a more precise prediction or hypothesis formulation in testing specific cognitive tasks; second, the narcissistic phenotype (as the sum of observable characteristics) delineated by commonly used narcissism inventories possibly capturing or emphasising different facets, which are not only partially non-overlapping in non-clinical cohorts, but also might not be extended to clinical manifestations of narcissistic traits, such as NaPD; thirdly, as with other cluster B personality features, narcissistic traits or behaviours might show considerable variability over time, calling for additional study of trait vs. state markers (Edershile et al., 2021). Given the limitations of our study, in particular its limited sample size, a critical discussion might serve to guide future studies in identifying inter-individual variation related to narcissism and its implications for a spectrum understanding.

In contrast to other personality dimensions, such as the borderline personality disorder phenotype, which includes neurocognitively relevant aspects of impulsivity (overlapping with attention-deficit disorders) and behavioural dysregulation (Sebastian et al., 2014) as well as neurobiologically established alterations (Davies et al., 2020; Grottaroli et al., 2020), there is no clearly delineated neural model for narcissism. While some studies have pointed to anterior insula grey matter reduction in NaPD (Schulze et al., 2013), some minor reductions also seem to be present in medial or lateral prefrontal areas (Schulze et al., 2013; Nenadic et al., 2015a), which are relevant for executive functions. Indeed, two mentioned studies in non-clinical subjects show a correlation with prefrontal cortical volumes (Mao et al., 2016) and prefronto-parietal functioning (Yang et al., 2015), respectively. While (lateral) prefrontal structural integrity or functioning is relevant to working memory (Leavitt et al., 2017), it has been shown to be related not only to updating of information in short-term storage, but also different aspects of such tasks, including decision-making and information updating (Hu et al., 2019), and also qualities of stimuli (e.g., self-related), which vary across cognitive assessments (Okon-Singer et al., 2015; Schurz et al., 2021; Yin et al., 2021). Variation in task design of paradigms tapping executive function (EF) can have considerable impact on the pattern of neural circuitry involved in each of these variants of EF performance, as shown in recent neuroimaging studies (Reineberg et al., 2018; Hu et al., 2019). Executive functions comprise different sets of cognitive abilities (Baddeley, 2012; Diamond, 2013; Cristofori et al., 2019), of which working memory might be considered one, but the tests used in the present study only covers the working memory subtests of WIE. Hence, variation in task design, such as emotional qualities of stimuli, might be relevant for associations of narcissistic features with cognition. In a recent study involving tasks related to inhibitory executive function and social pain, no correlation with narcissistic features was found (Buelow and Brunell, 2020), which might be consistent with the notion of a potential relation of narcissism and executive function being restricted to either particular aspects of such (complex) tasks or situational factors, i.e., aspects of temporal variability. A meta-analysis of ‘dark triad’ (i.e., Machiavellianism, narcissism and psychopathy) and general mental abilities did not support either negative nor positive correlations between narcissistic traits (measured with NPI or other inventories), but as a variety of general mental abilities with different cognitive domains was tapped, this further stresses the need for comparative studies across specific executive function tasks (O’Boyle et al., 2013).

The problem of different narcissistic phenotypes might add to this problem. While our study used the NPI as a well-established and commonly utilised self-report inventory for narcissistic traits, this might emphasise particular aspects of narcissism, such as grandiosity, social dominance or ‘expansive’ traits. Zhang and colleagues, who provided a more nuanced account of the effects of narcissistic traits on cognitive and motor functions (Zhang et al., 2020) differentiating adaptive from maladaptive narcissism. The former was defined to include authority and self-sufficiency (Zhang et al., 2020). However, the study of Zhang and colleagues showed marked differences to our in both subject selection, choice of an abbreviated NPI (NPI-16) and also its statistical approach. Given the direction of correlations, which was positive in these previous studies, and considered in our analysis approach, this also raises the problem in interpreting findings in the broader narcissistic spectrum: as most correlations in our study, albeit not significant, are indeed consistently positive, future research will have to consider whether different ranges of trait expression across the narcissism spectrum might show differences or ‘tipping points’, for example a tendency for improved cognitive function in low to moderately narcissistic subjects vs. those approaching or crossing diagnostic boundaries towards manifest disorder. In manifest NaPD, however, state-dependent effects during the course of the disorder might additionally impact on cognitive function, similarly to effects observed in borderline personality disorder (Davies et al., 2020).

As limited sample size is a major limitation of our study, our failure to detect a significant association calls for larger sample sizes (in addition to multiple narcissism inventories or phenotype aspects) to be studied in future research. While our initial sample size estimation was based on a more narrow, directed hypothesis testing, studies in larger samples would be needed to more fully test and understand the relations (including non-linear associations) between different facets of narcissism as well as different domains of executive functioning and different components of working memory; these aspects cannot be fully covered with our limited sample and range of cognitive tests. Even with our limited sample size, it is unlikely that larger effect sizes exist, so future studies might take into consideration that sufficient power is needed to detect smaller effect sizes, especially in non-clinical populations. The overall negative result of this study might thus prompt future studies to consider these aspects.

## Conclusion

In conclusion, our study fails to identify convincing evidence for an association of working memory function with narcissistic trait features in non-clinical subjects: despite some nominally significant associations, which were in line with our hypotheses, none of the correlations withstood correction for multiple comparisons. Yet, the trends are consistent with some recent findings in the literature and thus deserve additional studies, which might not only consider larger sample sizes, but also additional measures of narcissism (especially for the vulnerable trait facet). Such cognitive studies might provide a relevant link in the emerging neurobiological literature on narcissism, and similar to other cluster B personality features/disorders, might support establishing neural network models of personality differences.

## Data Availability Statement

Data available upon reasonable request, pertinent to ethics and legal regulations.

## Ethics Statement

The studies involving human participants were reviewed and approved by Ethikkommission der Medizinischen Fakultät der Friedrich-Schiller-Universität Jena, Jena, Germany. The participants provided their written informed consent to participate in this study.

## Author Contributions

IN conceived the study and its design, obtained the funding, supervised the recruitment, performed the statistical analysis of data, interpreted the data, and wrote the manuscript.

## Funding

Parts of this study were supported by a Junior Scientist Grant of the Friedrich-Schiller-University of Jena (DRM 210070/87 to I.N.) and EU (MRTN-CT-2006-035987 to I.N.).

## Conflict of Interest

The author declares that the research was conducted in the absence of any commercial or financial relationships that could be construed as a potential conflict of interest.

## Publisher’s Note

All claims expressed in this article are solely those of the authors and do not necessarily represent those of their affiliated organizations, or those of the publisher, the editors and the reviewers. Any product that may be evaluated in this article, or claim that may be made by its manufacturer, is not guaranteed or endorsed by the publisher.
